# Transcriptional profile of *Trichomonas vaginalis* in response to metronidazole

**DOI:** 10.1186/s12864-023-09339-9

**Published:** 2023-06-12

**Authors:** Yiting Xie, Ping Zhong, Wei Guan, Yanqing Zhao, Shuguo Yang, Yan Shao, Jian Li

**Affiliations:** 1 School of Basic Medical Science, Hubei University of Medicine, Shiyan, 442000 China; 2 Department of Outpatient, Taihe Hospital, Hubei University of Medicine, Shiyan, 442000 China

**Keywords:** *Trichomonas vaginalis*, Metronidazole, Transcriptome, Differentially expressed genes, Electron microscopy, Mechanism of action

## Abstract

**Background:**

Trichomoniasis caused by *Trichomonas vaginalis*, combined with its complications, has long frequently damaged millions of human health. Metronidazole (MTZ) is the first choice for therapy. Therefore, a better understanding of its trichomonacidal process to ultimately reveal the global mechanism of action is indispensable. To take a step toward this goal, electron microscopy and RNA sequencing were performed to fully reveal the early changes in *T. vaginalis* at the cellular and transcriptome levels after treatment with MTZ in vitro.

**Results:**

The results showed that the morphology and subcellular structures of *T. vaginalis* underwent prominent alterations, characterized by a rough surface with bubbly protrusions, broken holes and deformed nuclei with decreased nuclear membranes, chromatin and organelles. The RNA-seq data revealed a total of 10,937 differentially expressed genes (DEGs), consisting of 4,978 upregulated and 5,959 downregulated genes. Most DEGs for the known MTZ activators, such as pyruvate:ferredoxin oxidoreductase (PFOR) and iron-sulfur binding domain, were significantly downregulated. However, genes for other possible alternative MTZ activators such as thioredoxin reductase, nitroreductase family proteins and flavodoxin-like fold family proteins, were dramatically stimulated. GO and KEGG analyses revealed that genes for basic vital activities, proteostasis, replication and repair were stimulated under MTZ stress, but those for DNA synthesis, more complicated life activities such as the cell cycle, motility, signaling and even virulence were significantly inhibited in *T. vaginalis.* Meanwhile, increased single nucleotide polymorphism (SNP) and insertions - deletions (indels) were stimulated by MTZ.

**Conclusions:**

The current study reveals evident nuclear and cytomembrane damage and multiple variations in *T. vaginalis* at the transcriptional level. These data will offer a meaningful foundation for a deeper understanding of the MTZ trichomonacidal process and the transcriptional response of *T. vaginalis* to MTZ-induced stress or even cell death.

**Supplementary Information:**

The online version contains supplementary material available at 10.1186/s12864-023-09339-9.

## Background

Trichomoniasis caused by *Trichomonas vaginalis* is the most prevalent nonviral sexually transmitted disease worldwide. A recent report by the World Health Organization (WHO) estimated approximately 156 million new cases of trichononasis globally [1]. Usually, *T. vaginalis* infection can lead to variable clinical manifestations, ranging from asymptomatic carriers to patients with severe inflammation of the genital system in women or men [2]. Rather than these, it may also increase the risk of HIV [3] and cervical neoplasia [4] acquisition in women and probably result in several adverse pregnancy consequences [5]. Although infected males are widely asymptomatic and self-limited, the increased risk of nongonococcal urethritis [6], impaired infertility [7], prostate hyperplasia and even prostate cancer [8] associated with male trichomoniasis should not be ignored.

Metronidazole (MTZ), as one of the mainstay drugs for killing anaerobic bacteria, microaerophilic bacteria, and protozoa such as *Giardia lamblia*, *Entamoeba histolytica*, and *T. vaginalis*, has been widely used for the treatment of related infectious diseases in the clinic for several decades [9][10]. It is also considered the first choice for the systemic treatment of trichmononasis with an excellent cure rate, and there are no replacement drugs with superior advantages to date [2]. Therefore, a deeper understanding of its trichomonacidal process to ultimately fully reveal the global mechanism of action is indispensable and meaningful for the better and more accurate use of this drug against trichmononasis and even other pathogens. After decades of unremitting efforts by scientists, research on the mechanism by which MTZ kills *T. vaginalis* has made tremendous progress, however, it has not yet been fully illustrated [11].

As a prodrug, MTZ is inactive until it enters *T. vaginalis* and becomes activated after its nitro group is reduced to cytotoxic nitro radical anions [12, 13]. Previous studies revealed several MTZ activation-related molecules, such as pyruvate:ferredoxin oxidoreductase (PFOR), ferredoxin (Fd) [12, 14, 15] and the flavin enzyme thioredoxin reductase (TrxR) [16]. Studies also indicate that rapid responses of *T. vaginalis* exposure to MTZ occur in vitro, such as DNA synthesis interruption at approximately 30 min, cell division ceasing one hour later, motility stopping within one to two hours, and ultimately parasite death occurring within 5 to 8 h [17–19]. However, most studies about the mechanism of MTZ against *T. vaginalis* seemed to be focused on the activation of this drug and the final ending of the parasites. Regarding the influence of MTZ on *T. vaginalis* after MTZ treatment, but before *T. vaginalis* widely dies, especially how MTZ affects the gene expression of *T. vaginalis* post coincubation, there are limited documents.

Furthermore, there are frequently refractory or recurrent trichomoniasis cases reported in the clinic in recent years [20]. Investigate the reasons, downregulated genes or reduced activity of PFOR, Fd, flavin reductase and nitroreductase (NTR), in some aerobic or anaerobic resistant strains are probably closely related to it [14, 21–23]. However, changes in these genes cannot explain all cases of MTZ resistance [24]. The deeper and overall mechanism of MTZ resistance in trichomoniasis needs further investigation. Certainly, more explicit recognition of the MTZ action is beneficial for a better understanding of MTZ resistance.

Recently, transcriptome sequencing has provided an excellent tool to dissect the differentially expressed genes (DEGs) between different research conditions, which could supply global and important information reflecting the influence of one or more inducements on the target cells or tissues at the transcriptional level [25]. Therefore, the present study aims to reveal the changes in gene expression of *T. vaginalis* in response to MTZ at an early stage after MTZ treatment but before *T. vaginalis* widely dies depending on RNA-seq. The results will help us gain a more comprehensive understanding of the drug action model and the transcriptional response of *T. vaginalis*, or even other pathogens, to MTZ-induced stress and even cell death, which could also offer a clue to help understanding the resistant mechanism and develop new drugs.

## Results

### Structural changes in ***T. vaginalis*** after treatment with MTZ

According to Fig. S1A, the minimum lethal concentration (MLC) of MTZ on TV-THS1 was 12.5 µg/ml, lower than the resistance threshold (50 µg/ml) [26]. Therefore, TV-THS1 was considered to be a sensitive strain. The drug concentration at which approximately half of the parasites compared to the control (MTZ = 0 µg/ml) remained after treatment with MTZ for 3 h was 25 µg/ml (Fig. S1B). Therefore, *T. vaginalis* treated with MTZ at 25 µg/ml for 3 h was chosen for morphology and subcellular structure analysis and RNA-seq.

**Fig. 1:**
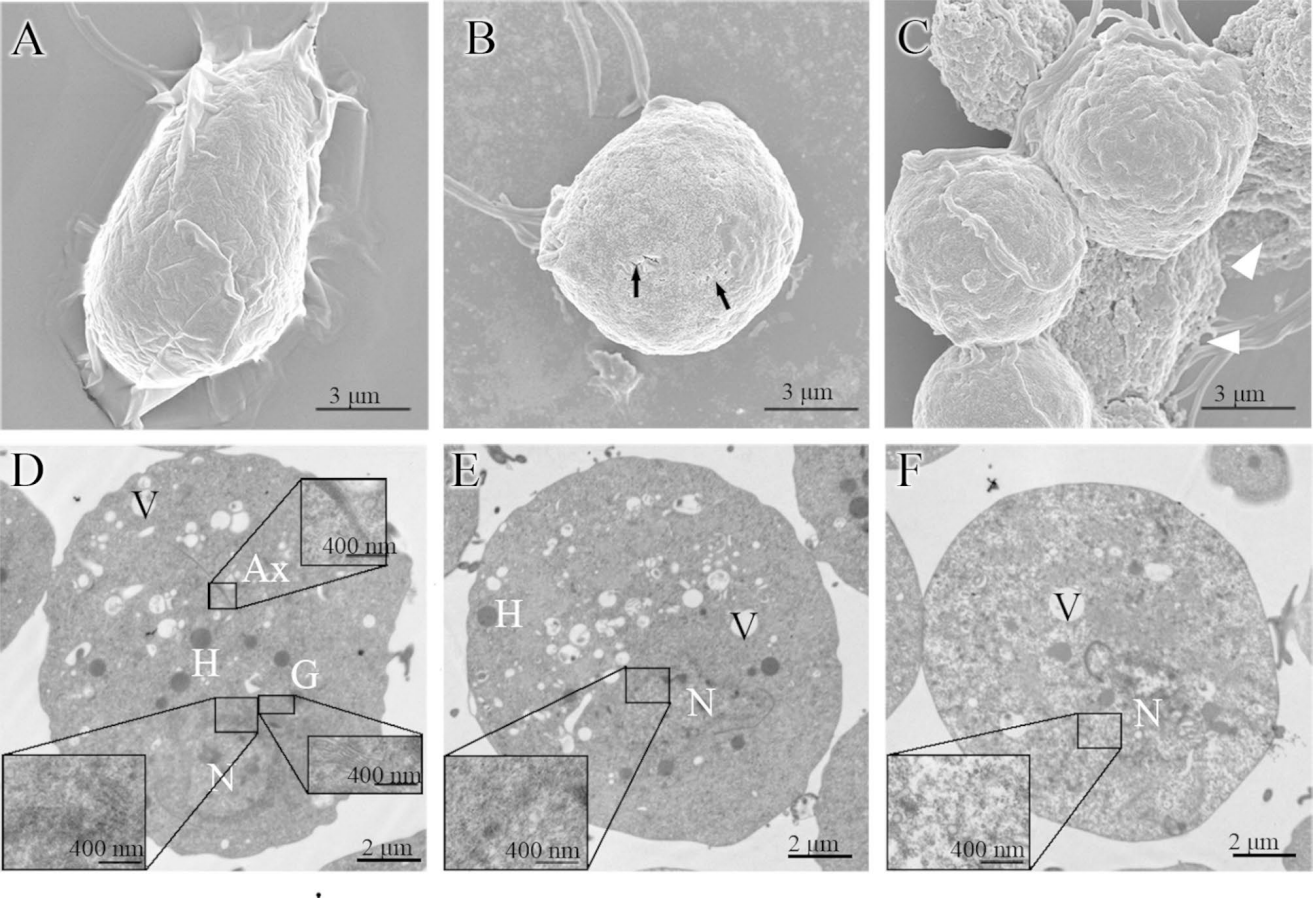
Structural changes in *T. vaginalis* after treatment with MTZ. (**A**) SEM image of untreated *T. vaginalis*. (**B** and **C**) SEM image of *T. vaginalis* after treatment with MTZ. (**D**) TEM image of untreated *T. vaginalis*. (**E** and **F**) TEM image of *T. vaginalis* after treatment with MTZ. N, nucleus; H, hydrogenosome; Ax, axostyle; V, vacuole; G, Golgi complex. The black arrow and white triangle indicate the break in the cytomembrane of *T. vaginalis* and residual debris after treatment with MTZ, respectively

Observation by scanning electron microscopy showed that the trophozoites of normal *T. vaginalis* were oval or pear-shaped. There were folds of different depths on the cell surface, and the membrane was clear (Fig. 1A). After MTZ treatment for 3 h, *T. vaginalis* was still ovoid, but the surface was rough, and there were many bubbly protrusions and small depressions of varying sizes. There were also obvious broken holes on the surface of the cell membrane with off-white granular substances attached to the surface of the trophozoites (Fig. 1B). For the severely damaged parasites, the cell membrane was completely broken, leaving the cauliflower-like cytoplasmic contents (Fig. 1C).

Transmission electron microscopy presented that normal *T. vaginalis* had an intact cell membrane. The nuclear membrane was also intact with abundant nuclear chromatin inside. The endoplasmic reticulum, Golgi apparatus, and axis column were all clearly visible (Fig. 1D). After treatment with MTZ for 3 h, *T. vaginalis* underwent prominent alterations. The nucleus was deformed in morphology. Part of the nuclear membrane disappeared, and the boundary between the nucleus and cytoplasm was blurred (Fig. 1E). The Golgi and endoplasmic reticulum were also blurred and even disappeared. For some trophozoites, the chromatin in the nucleus decreased, or the electron density in the entire cell was significantly reduced (Fig. 1F). Furthermore, some trophozoites were obviously disrupted, leaving only a network structure. However, compared with the control (untreated group), the morphology and number of hydrogenases and vacuoles did not change significantly.

### General property and quality assessment of transcriptome sequencing

First, the quality of sequencing data was assessed, which consisted of the data output statistics, base content across all bases, quality scores across all bases, and sequence GC content. As shown in Table 1, the sequencing finally produced an average of 20,945,600 and 19,660,581 clean read pairs with extremely low error rates in the MTZ-treated group (Q20: 96.9%) and untreated group (Q20: 97.1%), respectively. The highly matched base pairs (A-T, C-G), good quality for each base, and the approximately 45% GC content all demonstrated the extremely high quality and accuracy of the current sequencing data (Fig. S2). Of these data sets (Table S1), 36,469,263 (87.07%, MTZ treated) and 35,284,989 (89.73%, untreated) clean reads were mapped to the reference genome of *T. vaginalis*. A total of 22,810 (MTZ-treated) and 23,208 (untreated) expressed genes were clustered by the fragments per kilobase per million bases (FPKM) values for five intervals. Of these, gene numbers within the FPKM > 1 intervals all dramatically deceased after MTZ treatment (Table S2).

**Table 1:** The data output statistics

Sample	Clean Reads Pairs	Clean base (bp)	Length	Q20 (%)	Q30 (%)	GC (%)
**TV-THS1-G49**	18,306,176	5,491,852,800	150;150	96.9;97.1	91.9;93.1	44.9;44.8
**TV-THS1-G50**	21,883,872	6,565,161,600	150;150	97.0;97.4	92.1;93.6	45.2;45.1
**TV-THS1-G51**	18,791,695	5,637,508,500	150;150	97.0;97.3	92.1;93.5	44.5;44.3
**TV-THS1-G49-MTZ**	20,581,896	6,174,568,800	150;150	96.7;96.8	91.6;92.7	45.5;45.3
**TV-THS1-G50-MTZ**	21,319,745	6,395,923,500	150;150	96.8;97.2	91.8;93.4	44.9;44.7
**TV-THS1-G51-MTZ**	20,935,160	6,280,548,000	150;150	97.0;97.0	92.3;92.9	44.6;44.4

**Fig. 2:**
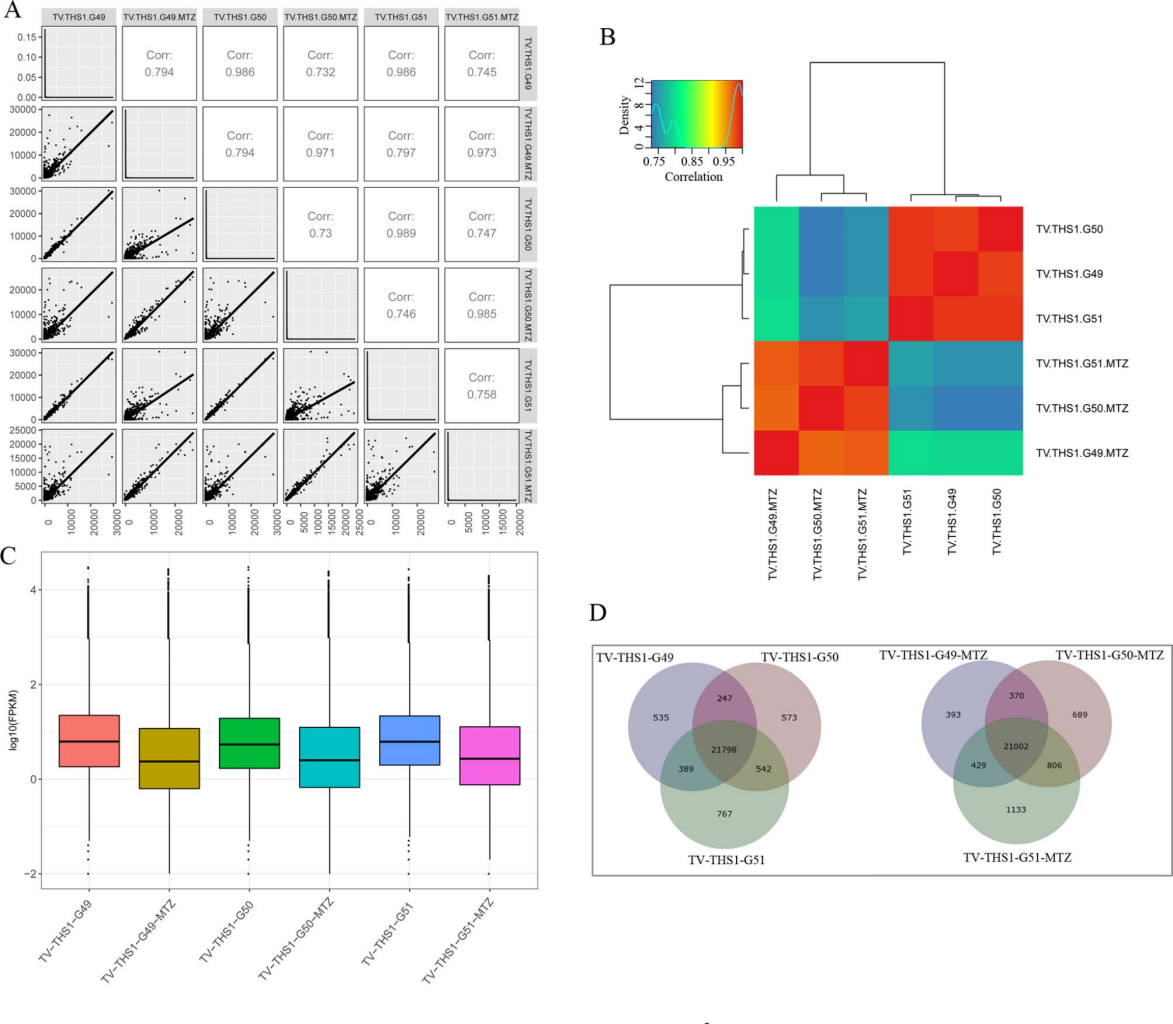
Quality assessment of transcriptome sequencing. (**A**) Correlation analysis of gene expression levels between samples. The closer the correlation coefficient is to 1, the higher the similarity of the expression patterns between samples. (**B**) Heatmap of gene expression levels between samples. (**C**) Statistical distribution of gene FPKM. (**D**) Overlap of genes within the three repeat samples in the same group by Venn diagram

High coverage depth and homogenization in data sets revealed the high quality of the RNA-seq in the untreated group, but RNA degradation appeared in the MTZ-treated group (Fig. S3). The saturability differentiation of an expression level less than 15% guaranteed quantitative accuracy (Fig. S3). The high repeatability and correlation of gene expression within the three repeats in both the untreated and MTZ-treated groups further manifested the experimental reliability and reasonability of sample selection (Fig. 2A and B). Additionally, the density distribution of genes all disclosed high consistency within the three repeats in each group and total downregulated expression in MTZ-treated samples (Fig. 2 C and 2D).

### Analysis of differentially expressed genes

**Fig. 3:**
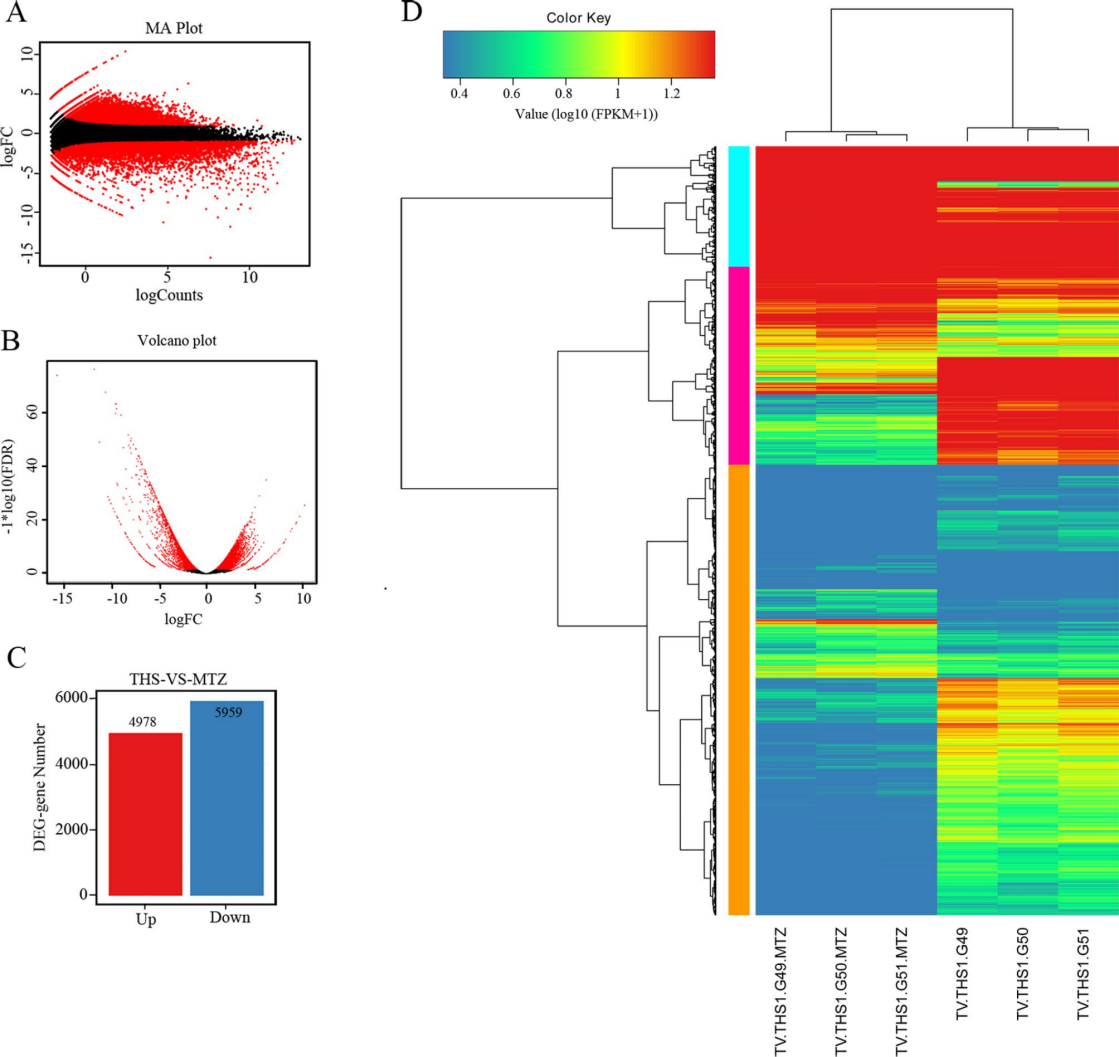
Screening of DEGs and expression level clustering. (**A**) Distribution of DEGs by MA plot. (**B**) Distribution of DEGs by volcano plot. (**C**) Statistics of the upregulated and downregulated DEGs. In the generated MA plot and volcano plot, black dots represent genes that do not meet the screening criteria, and red dots are DEGs according to the threshold. (**D**) Heatmap of DEGs in *T. vaginalis* treated and untreated with MTZ. The color scale (upper left) indicates the relative expression values (log_10_ (FPKM + 1))

To globally assess the quantitative changes in the MTZ-treated transcriptome, the DEGs (red dots) were screened out by edgeR, and the selective condition was defined as false discovery rate (FDR) < 0.05 and Log_2_ Fold Change (MTZ-treated/MTZ-untreated) (Log_2_ FC) > 1 or Log_2_ FC <-1. The MA plot (Fig. 3A) and volcano plot (Fig. 3B) visually show that more genes were inhibited than stimulated in *T. vaginalis* after MTZ treatment. Only a relatively small portion of the genes were not differentially expressed (black dots). Statistically, a total of 10,937 genes were differentially expressed, including 4,978 upregulated and 5,959 downregulated genes (Fig. 3C). The heatmap and clustering tree revealed a distinct gene expression pattern between the MTZ treated and untreated groups, but similar within a group (Fig. 3D). The hierarchical clustering revealed three clusters of genes.

### Differential expression of known MTZ activators

Of these genes (Table 2), three of the five annotated genes for PFOR were differentially expressed, with one upregulated approximately 4.0-fold and two downregulated approximately 3.0-fold and 2.4-fold, respectively. Four genes for the 4Fe-4 S binding domain-containing protein showed differential expression, with only one upregulated approximately 2.2-fold and the remaining three downregulated approximately 3.0- to 10.3-fold. However, six annotated genes for Fd did not change significantly. Additionally, one of three genes mapped to thioredoxin reductase, an alternative MTZ activator, was significantly upregulated by approximately 3.8-fold. Five of six DEGs encoding nitroreductase family proteins were significantly upregulated by approximately 9.2- to even 739.3-fold. Meanwhile, 24 genes annotated to the flavodoxin-like fold family proteins were differentially expressed, including two downregulated approximately 3.0-fold and 22 upregulated approximately 2.1- to even 685.0-fold (Table 3).

**Table 2:** Differential expression of the previously identified genes involved in MTZ activation

GenBank Accession Numbers	Gene Annotation	log_2_FC (MTZ-treated/ MTZ-untreated )
EAY02588.1	Pyruvate:ferredoxin oxidoreductase E	2.04
EAX94158.1	Pyruvate:ferredoxin oxidoreductase C	-1.26
AAA85495.1	Pyruvate:ferredoxin oxidoreductase proprotein	-1.61
EAY08460.1	4Fe-4 S binding domain containing protein	1.13
EAY19262.1	4Fe-4 S binding domain containing protein	-3.37
EAY03354.1	4Fe-4 S binding domain containing protein	-1.57
EAY22303.1	4Fe-4 S binding domain containing protein	-1.90
EAY04700.1	Thioredoxin reductase	1.91
EAY14310.1	Nitroreductase family protein	3.415058
EAY07485.1	Nitroreductase family protein	3.203646
EAY04224.1	Nitroreductase family protein	9.530239
EAX89456.1	Nitroreductase family protein	3.535082
EAY17166.1	Nitroreductase family protein	7.074432
EAY16021.1	Nitroreductase family protein	-1.91617

**Table 3:** Differential expression of genes for Flavodoxin-like fold

GenBank Accession Numbers	Gene Annotation	log_2_FC (MTZ-treated/ MTZ-untreated )
EAY20871.1	Flavodoxin-like fold family protein	3.150
EAX89362.1	Flavodoxin-like fold family protein	2.315
EAX89359.1	Flavodoxin-like fold family protein	2.011
EAX97338.1	Flavodoxin-like fold family protein	3.726
EAX98223.1	Flavodoxin-like fold family protein	2.010
EAX91048.1	Flavodoxin family protein	1.362
EAY11224.1	Flavodoxin-like fold family protein	2.293
EAY07033.1	Flavodoxin-like fold family protein	1.835
EAY00354.1	Flavodoxin-like fold family protein	3.087
EAY00346.1	Flavodoxin-like fold family protein	-1.642
EAX83483.1	Flavodoxin-like fold family protein	1.925
EAY15747.1	Flavodoxin-like fold family protein	4.572
EAY05660.1	Flavodoxin family protein	1.074
EAY23002.1	Flavodoxin-like fold family protein	-1.629
XP_001306578.1	Flavodoxin-like fold family protein	5.294
XP_001307810.1	Flavodoxin-like fold family protein	5.682
XP_001303716.1	Flavodoxin-like fold family protein	6.716
XP_001303721.1	Flavodoxin-like fold family protein	6.460
XP_001322702.1	Flavodoxin-like fold	3.330
XP_001317528.1	Flavodoxin-like fold	6.704
XP_001317526.1	Flavodoxin-like fold	3.298
XP_001306204.1	Flavodoxin-like fold	9.420
XP_001306582.1	Flavodoxin-like fold	4.015
XP_001319426.1	Flavodoxin-like fold	3.763

### GO and KEGG enrichment analysis of the DEGs

**Fig. 4:**
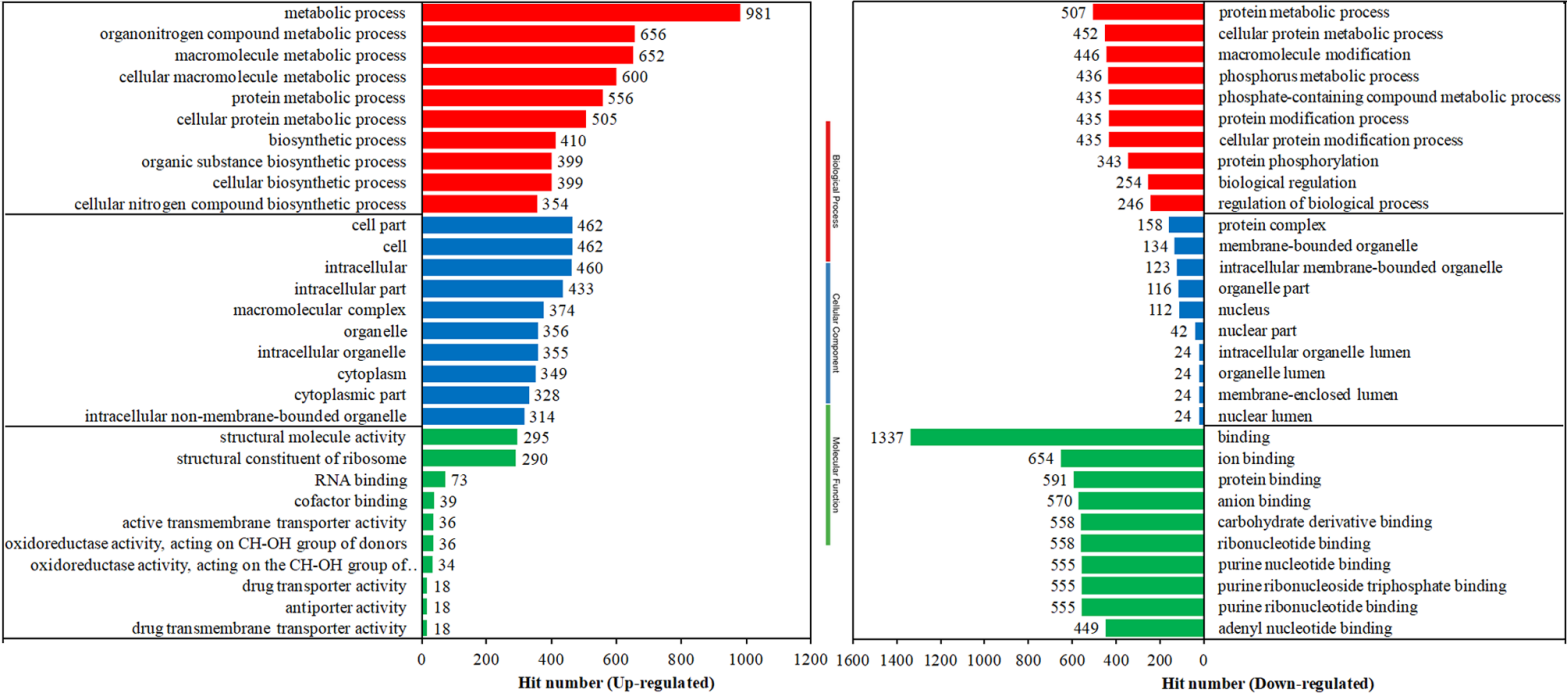
GO enrichment of DEGs. The hit number of the top 10 significantly enriched (Q-value < 0.05) terms in each primary catalog, including biological process, cellular component and molecular function, in the upregulated and downregulated DEGs

To further explore the changes in DEGs of *T. vaginalis* exposed to MTZ, GO and KEGG analysis were performed. The significantly enriched (Q value < 0.05) upregulated DEGs consisted of 74 GO terms in biological processes, 25 terms in cellular components and 31 terms in molecular functions (Table S3). Accordingly, the downregulated DEGs were significantly enriched in 78 biological process terms, 31 cellular component terms and 43 molecular function terms (Table S4). As shown in Fig. 4, the top 10 significantly enriched terms in each primary displayed that genes for metabolism and biosynthetic processes, intracellular components (especially for organelles and cytoplasm), structural molecules, and oxidoreductase activity were specifically upregulated together with genes involved in transmembrane-related transport, such as drug transmembrane transporters. In contrast, a relatively smaller number of genes involved in metabolic processes and organelles/organellar lumen were significantly downregulated. Instead, large numbers of genes contributing to macromolecule/protein modification, biological regulation, the nucleus, and bandings of various intracellular molecules, especially nucleotide binding, were significantly inhibited.

**Fig. 5:**
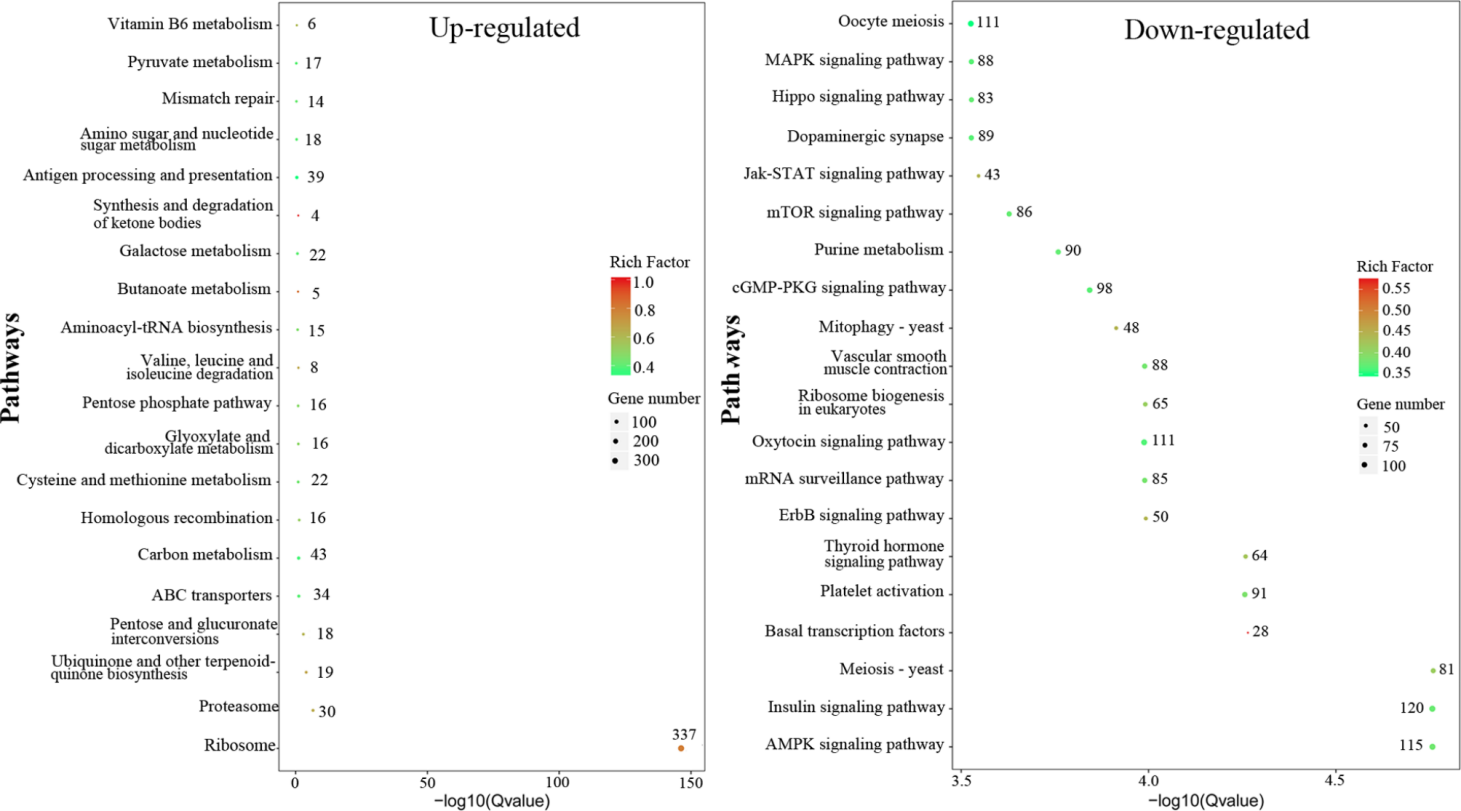
KEGG enrichment of DEGs. The top 20 most significantly enriched pathways (Q-value < 0.05) were selected for drawing the enrichment scatter diagram of DEGs. For the upregulated DEGs, only the top 15 most enriched pathways were significant

However, KEGG analysis revealed many more significantly enriched downregulated pathways than upregulated pathways. As shown in Fig. 5, among the 15 significantly upregulated pathways, genes were mainly mapped to ribosome (337 out of 421), aminoacyl-tRNA biosynthesis (15 out of 33) and proteasome (30 out of 46). In accordance with the upregulated GO terms, a total of 173 genes were significantly enriched in metabolism-related pathways involving amino acid carbon, carbohydrate, lipid, cofactors and vitamin. Interestingly, 16 out of the 31 genes involved in homologous recombination during replication and repair and 34 out of the 88 genes involved in ABC transporters were significantly upregulated. For the top 20 significantly downregulated pathways (Fig. 5), genes were widely mapped to the following various levels: translation (e.g., mRNA surveillance pathway), nucleotide metabolism (e.g., purine metabolism), cell growth and death (e.g., cell cycle, meiosis), cell motility (e.g., regulation of actin cytoskeleton), cellular community-eukaryotes (e.g., adherens junction, focal adhesion, gap junction), transport and catabolism (e.g., mitophagy, autophagy), signal transduction in environmental information processing sets and organismal systems (e.g., circulatory system, endocrine system, environmental adaptation, excretory system, immune system, nervous system and sensory system). Further analysis of the other 37 significantly enriched KEGG pathways showed that many genes involved in system regulation, transcription, translation, signal transduction, transport, catabolism, community, cell growth and death, as well as those involved in various signaling pathways, cellular junctions and the cell cycle, were dramatically downregulated (Table S5).

### Single nucleotide polymorphism (SNP) and insertion - deletions (indel) analysis

After MTZ treatment, the total SNPs increased but were not significant (Table 4). However, a more interesting finding in our study showed that dramatically increased indels (from 568 to 2161, *P* = 0.0317), especially heterozygous indels (from 289 to 1939, *P* = 0.0275), appeared in the transcripts after MTZ treatment. Further analysis showed that the main types of these indels were deletions, and most of them were G/C deletions. For both the untreated (62%) and MTZ treated groups (56%), most SNPs appeared in exonic regions. However, SNPs were significantly increased in other regions including the 3’UTR, introns and [upstream; downstream] (3 kb region of upstream from transcription start site; 3 kb region of downstream from the transcription termination site) after MTZ treatment. Similarly, the exonic region contained the most indels for both groups. By MTZ treatment, indels in regions of downstream (3 kb region of downstream from the transcription termination site), exonic, intronic and upstream (3 kb region of upstream from transcription start site) were all significantly increased (Fig. S4).

**Table 4:** Statistics of the SNP and Indel

Sample	Total SNP	Homozygosis SNP	Heterozygosis SNP	Total Indel	Homozygosis Indel	Heterozygosis Indel
**TV-THS1-G49**	1,257	251	1,006	536	254	282
**TV-THS1-G50**	1,359	287	1,072	591	278	313
**TV-THS1-G51**	1,420	296	1,124	577	304	273
**Average**	1,345	278	1,067	568	279	289
**TV-THS1-G49-MTZ**	1,326	279	1,047	1,729	205	1,524
**TV-THS1-G50-MTZ**	1,426	283	1,143	2,036	217	1,819
**TV-THS1-G51-MTZ**	1,694	300	1,394	2,718	245	2,473
**Average**	1,482	287	1,195	2,161	222	1,939

### Confirmation of gene expression by RT‒qPCR

**Fig. 6:**
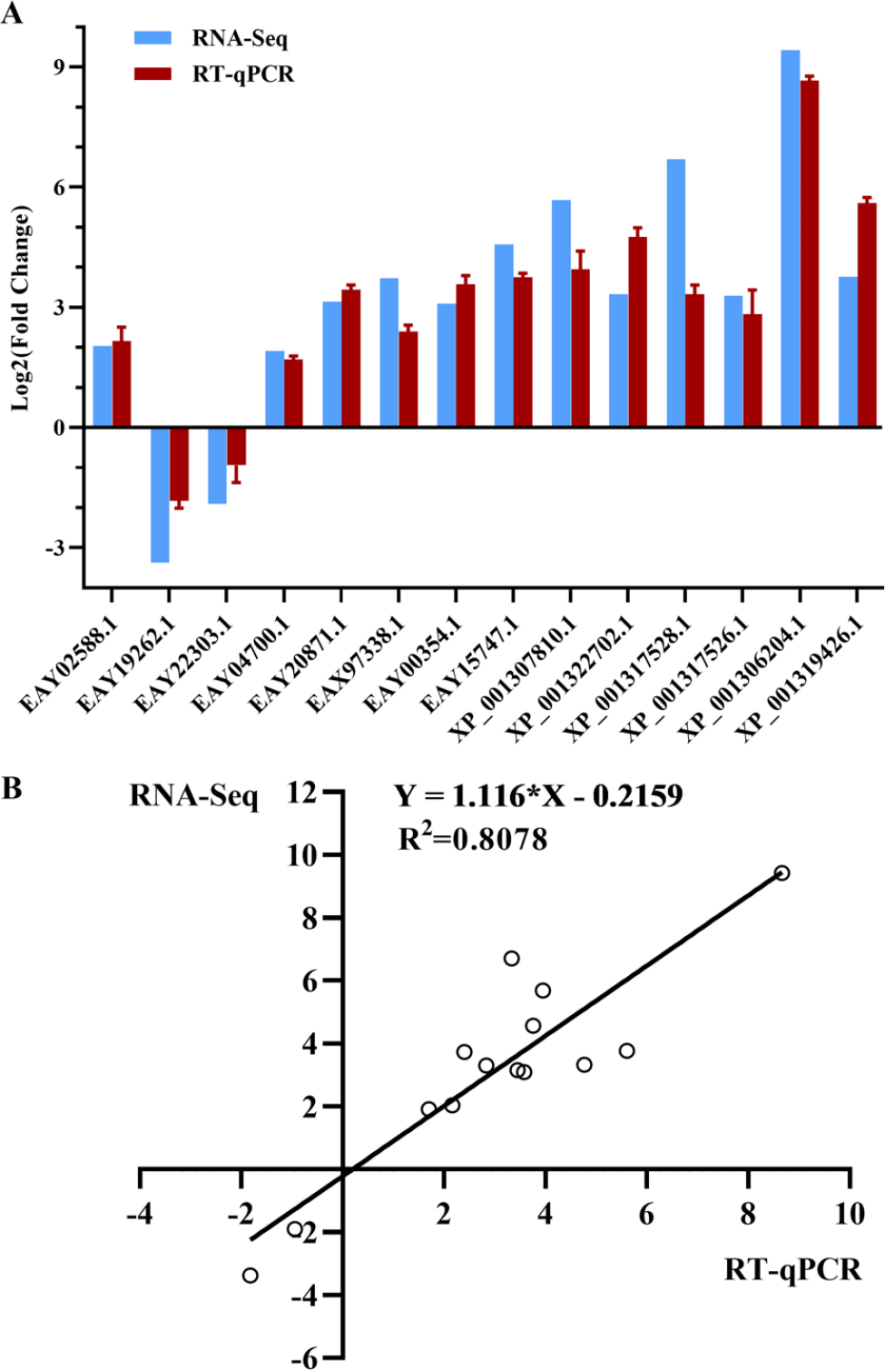
Validation of DEGs related to known MTZ activators by RT‒qPCR. (**A**) Validation of 14 significant DEGs between MTZ-treated and untreated samples by qPCR using the Log_2_ FC method. The relative RNA quantity of each target gene was normalized against the expression levels of actin with the comparative 2^−ΔΔCT^ method. Three technical replicates were set up for each biological replicate. Values of RT‒qPCR shown are the mean with SD. (**B**) Correlation measured by log_2_ FC between RT‒qPCR (x-axis) and RNA-Seq (y-axis)

To validate the RNA-seq results, expression level of 14 significant DEGs closely related to the known MTZ activators were relatively quantified by RT‒qPCR (Fig. 6A), including PFOR E (EAY02588.1), 4Fe-4 S binding domain containing protein (EAY19262.1 and EAY22303.1), thioredoxin reductase (EAY04700.1) and ten flavodoxin-like fold family proteins (EAY20871.1, EAX97338.1, EAY00354.1, EAY15747.1, XP_001307810.1, XP_001322702.1, XP_001317528.1, XP_001317526.1, XP_001306204.1 and XP_001319426.1). All the primers for target genes and reference gene [27] are shown in Table S6. The results of RT‒qPCR showed that the selected genes were significantly differentially expressed, and their upregulated or downregulated trends were consistent with those obtained by RNA-Seq. Additionally, a high correlation (R^2^ = 0.8078) measured by Log_2_ FC between RNA-Seq and RT‒qPCR was observed (Fig. 6B), indicating the reliability of DEGs obtained from transcriptome sequencing.

## Discussion

Revealing the transcriptional changes in *T. vaginalis* after MTZ treatment, but before *T. vaginalis* widely dies, can assist in a deeper understanding of the MTZ trichomonacidal process and the transcriptional response of *T. vaginalis* to MTZ-induced stress or even cell death. After MTZ treatment for 3 h, variations in the morphology and subcellular structures of *T. vaginalis* were observed. RNA-seq revealed more downregulated DEGs than the upregulated ones. Of these, most DEGs referring to the well-known MTZ activators, such as PFOR and iron-sulfur binding domain, were significantly inhibited, but gene sets annotated to thioredoxin reductase, nitroreductase family proteins and flavodoxin -like fold family proteins, the other possible alternative MTZ activators, were dramatically stimulated. GO and KEGG analyses revealed that genes for basic vital activities, proteostasis, replication and repair were stimulated under MTZ stress, but those for DNA synthesis, more complicated life activities such as the cell cycle, motility, signaling and even virulence were significantly inhibited in *T. vaginalis.*

Of the 10,937 DEGs, 4,978 upregulated and 5,959 downregulated DEGs were discovered in *T. vaginalis*, suggesting that more genes were significantly downregulated in *T. vaginalis* in response to MTZ-induced stress. This is different from a previous study in which 14,072 DEGs were found, with 8432 upregulated and 6270 downregulated [28]. A possible cause of the difference should be the diversity in experimental materials and conditions between the two studies, such as *T. vaginalis* isolates, MTZ concentrations and the time for treatment. This, accompanied by the inherent variability of the transcriptome, indicated that the results of one transcriptome-based study would be theoretically difficult to resolve the target problem in one study. In other words, the shared findings among different studies probably have greater significance.

The DEGs encoding PFOR and 4Fe-4 S binding domain-containing proteins were almost all downregulated by 2.4- to even 10.3-fold, except for two that were upregulated. PFORs from *T. vaginalis* are known as homodimers of 240 to 280 kDa containing 2[4Fe-4 S] clusters [29]. A previous study concluded that a strong correlation existed between the presence of PFOR activity and MTZ among different microorganisms [29]. Previous studies also reported that genes for PFOR were significantly downregulated in resistant isolates of *T. vaginalis* [28] and *Giardia lamblia* [30]. Therefore, as the key proteins for MTZ activation, the wide downregulation of PFOR was likely to be a self-rescue response at the early stage. However, one of three genes mapped to thioredoxin reductase and five of six DEGs encoding nitroreductase family proteins were significantly upregulated by approximately 3.8-to to even 739.3-fold. Thioredoxin reductase (with nitroreductase activity) and nitroreductase have been reported to play roles in activating MTZ against *T. vagianlis* depending on an alternative theory [16]. A “covalent adduct” between MTZ with thioredoxin reductase and other enzymes can be formed, leading to the failure of thioredoxin activation and then blocking thioredoxin peroxidase from reducing H_2_O_2_, which is cytotoxic. This, perhaps, is an important mechanism of action of MTZ other than DNA disruption [16, 20]. Interestingly, studies have confirmed that nitroreductase gene is also an important sign of MTZ resistance, which mainly refers to SNPs in two nitroreductase genes (*ntr4*_*Tv*_ and *ntr6*_*Tv*_). These SNPs introduced nonsense mutations, leading to stop codons in *ntr4Tv* and *ntr6Tv* associated with MTZ resistance [20, 21]. Therefore, their upregulation in the present study may further demonstrate the important alternative roles of these enzymes in MTZ action. Additionally, another family including 24 DEGs was annotated to flavodoxin-like fold sets, with 22 upregulated approximately 2.1- to even 685.0-fold. Flavodoxin - dependent MTZ reduction has been reported in *Helicobacter pylori* [31], indicating a possible functional prediction of these flavodoxin-like fold genes in *T. vaginalis*. Actually, a study proved that flavodoxin-like proteins in *T. vaginalis*, as functionally redundant electron donors with Fd (no differential expression in the present study), were indeed capable of activating MTZ [32]. Its wide and high upregulation presents an interesting target involved in MTZ activation that deserves further concern, especially in MTZ-resistant *T. vaginalis* isolates.

From the significantly enriched GO terms, genes responsible for metabolism, intracellular components, structural molecules and oxidoreductase activity were the most upregulated, indicating that responses supporting basic vital activity were stimulated under MTZ stress. Instead, large numbers of genes contributing to macromolecule/protein modification and biological regulation, especially cell nuclear and nucleotide binding, were inhibited. This was in accordance with findings in a previous study showing that most genes involved in nucleotide metabolism were dramatically suppressed in MTZ-sensitive parasites after MTZ treatment [28]. These results, combined with the broken nuclear membrane and decreased chromatin observed by TEM in the present study, further verify the DNA damage-dependent mechanism of MTZ.

In KEGG analysis, the significantly enriched upregulated genes were mainly involved in the basic life process, similar to the GO analysis. However, three special pathways were also stimulated under MTZ stress: proteasome for folding, sorting degradation, homologous recombination for replication and repair, and ABC transporters. Of these, all 14 standard proteasome subunits of core particles for the 20 S proteasome were significantly upregulated after MTZ treatment by approximately 2- to 4-fold. The 20 S proteasome, as a large protease complex consisting of seven α subunits (α1 to α7) and seven β subunits (β1 to β7), can widely degrade intracellular proteins in all eukaryotes [33]. This is meaningful for proteostasis because the accumulation of unfolded or misfolded proteins in the endoplasmic reticulum lumen might be harmful to cells [34]. Short-term incubation of *T. vaginalis* with a proteasome inhibitor leads to autophagy of parasites, implying its important function in *T. vaginalis* survival [35, 36]. Upregulation of the replication and repair gene pathway is also important for *T. vaginalis* survival under emergency conditions. For membrane transport, genes in ABC transporter pathways with a rich factor of 38.6% (34/88) were upregulated. Of these, a total of 18/34 genes were enriched in the ATP-binding cassette, subfamily B (MDR/TAP) and subfamily A (ABC1), and member 3 pathways with high Log_2_ FC even reached up to 8.0. Interestingly, these two ATP-binding cassette subfamilies were all reported to be multidrug resistance-associated proteins in humans [37, 38]. Therefore, the upregulation of ABC transporters may be a self-saving route for *T. vaginalis* to resist damage by MTZ.

Purines and pyrimidines are essential precursors for nucleotide and nucleic acid synthesis. However, *T. vaginalis* lacks the enzymes to synthesize de novo nucleotides, making it an obligate parasite reliant on salvage pathways. Purine salvage is mediated by some nucleoside phosphorylases and kinases [39]. KEGG data in the present study revealed that 90 genes in the purine metabolism pathway were significantly suppressed. This indicated that the normal nucleic acid metabolic pathway was sharply disturbed, which may be another mechanism by which MTZ functions. Widely downregulated transcripts of mRNA surveillance pathways (85/222) indicate the collapse in fidelity and quality of mRNA. This is consistent with an interesting finding in our study that a dramatic increase in indels (from 568 to 2161), especially the G/C deletion, was observed in the transcripts after MTZ treatment. Previous studies revealed that the nitro radical of MTZ is partial to targeting sections of DNA rich in the thymine and adenine residues (A + T) [13, 19]. In genome of *T. vaginalis*, approximately 71% of the A + T content explained the specificity and sensitivity of MTZ for this parasite [13]. The significantly increased deletion of G/C, may imply another mechanism of MTZ actions. In which, a higher proportion of A + T residues were left and enriched after G/C deletion at the gene level, resulting in a more severe impairment in these regions. Of course, this speculation needs the direct and reliable evidence. Additionally, the increased SNPs and indels in specific regions may introduce more coding variations, such as missense mutation or nonsense mutation. For example, the formation of premature stop codons in genes due to SNPs or indels, especially for those closely related to the drug’s action, perhaps leads to the inactivation of key enzymes and then drug resistance appears [24, 40, 41]. As reported in another study, 72 SNPs in *T. vaginalis* were proven to be associated with MTZ resistance [24].

The downregulation of cell meiosis and cell cycle genes indicated that the cell cycle of *T. vaginalis* was rapidly disrupted and may even arrest. This is in accordance with our observation of the structure of *T. vaginalis* by SEM/TEM and the previous finding that DNA synthesis could be inhibited within 30 min and *T. vaginalis* died within 5 h after incubation with MTZ in vitro [17]. It is also worth mentioning that the wide downregulation of genes for actin cytoskeleton regulation should be responsible for the rapid decrease in cell motility. At 3 h postincubation with MTZ in the present study, almost all *T. vaginalis* had stopped moving. The downregulated DEGs in adhesion and junctions suggested that the adhesion-based virulence of *T. vaginalis* was reduced. After all, motility and adhesion to host cells are pivotal steps in the pathogenesis of *T. vaginalis* [42]. Remarkably, genes for many signaling pathways were downregulated. In brief, the genes for purine metabolism, mRNA surveillance, cell cycle, various signaling pathways, motility and adhesion were inhibited, indicating that widely transcriptional disorders have appeared from DNA synthesis to more complicated life activities and even virulence in *T. vaginalis.*

Although our study revealed early changes in *T. vaginalis* at the cellular and transcriptome levels after treatment with MTZ for 3 h in vitro, there were also limitations in the study. For example, dynamic observation at more time points, including the early, middle, and universal dying stages, and the involvement of more different strains of *T. vaginalis* should have more advantages for better understanding the trichomonacidal process of MTZ.

## Conclusions

**Fig. 7:**
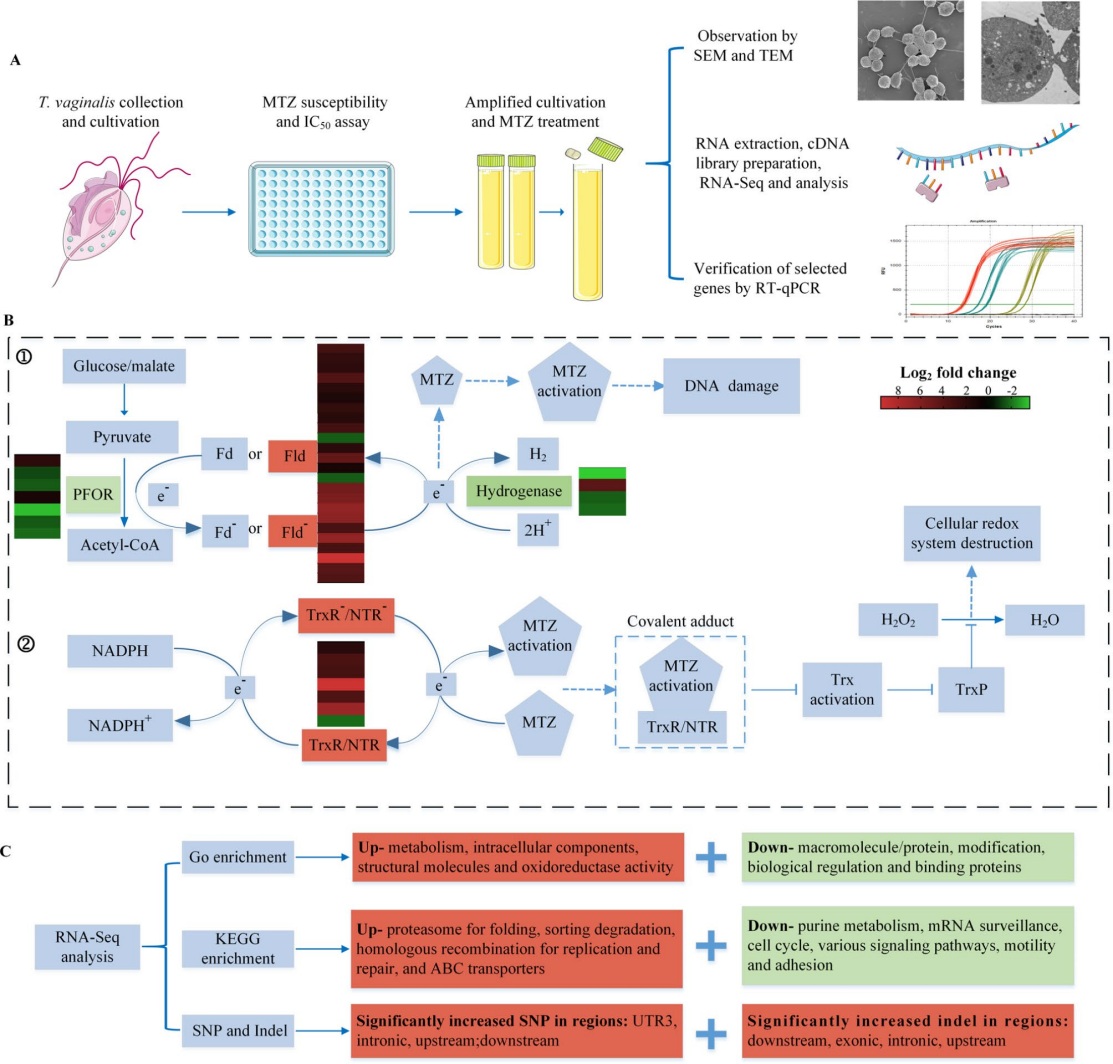
The flow chart and main findings of the present study. (**A**) Flow chart of the study design. (**B**) The differential expression of key genes responsible for MTZ activation involved in two possible pathways: ① Flavodoxin, as functionally redundant electron donors with Fd, accepts electrons from the process of oxidative decarboxylation of pyruvate catalyzed by PFOR and then transfers electrons to MTZ, resulting in MTZ activation to form a cytotoxic nitro radical anion. The latter can cause DNA damage and death in *T. vaginalis*. ② TrxR/NTR can use NADH as the electron donor to reduce MTZ. The activated MTZ then interacts with TrxR/NTR to form a covalent adduct, which would cause the failure of thioredoxin activation and then block thioredoxin peroxidase from reducing H_2_O_2,_ leading to the disrupted cellular redox system of *T. vaginalis*. (**C**) The major changes revealed by GO enrichment, KEGG enrichment, SNP and indel. In the heatmaps of (B), red and green indicate Log_2_ (fold change) of the upregulated and downregulated genes, respectively. Abbreviations: [PFOR] Pyruvate:ferredoxin oxidoreductase, [Fd] Ferredoxin, [Fld] Flavodoxin, [MTZ] Metronidazole, [NADPH] Nicotinamide adenine dinucleotide phosphate, [TrxR] Thioredoxin reductase, [NTR] Nitroreductase, [Trx]Thioredoxin, [TrxP] Thioredoxin peroxidase

The present study focuses on the total changes in the morphology, subcellular structures and transcriptome of *T. vaginalis* after MTZ treatment in vitro (Fig. 7A). The results revealed evident nuclear and cytomembrane damage beyond other changes and multiple changes in *T. vaginalis* at the transcriptional level. Specifically, key genes for MTZ activation were highly concerned (Fig. 7B). Other genes for basic vital activities, proteostasis and replication and repair were stimulated under MTZ stress, but those for DNA synthesis, more complicated life activities and even virulence were significantly inhibited in *T. vaginalis* (Fig. 7C). Meanwhile, SNPs and indels in specific regions of genes were increased after MTZ treatment (Fig. 7C). These data will offer a meaningful foundation for a deeper understanding of the MTZ trichomonacidal process and the transcriptional response of *T. vaginalis* to MTZ-induced stress or even cell death, which potentially takes a step toward ultimately revealing the global mechanism of MTZ action and then helps understanding the resistant mechanism.

## Materials and methods

### Ethical statement

The *T. vaginalis* strain TV-THS1 used in this work was isolated from a female patient undergoing routine examination in Taihe Hospital (Shiyan, China). The leucorrhea specimen was obtained with cotton swabs, kept in normal saline and passaged for 48 generations before being used in vitro. Ethical approval for this study was obtained from the Medical Ethics Committee of the Hubei University of Medicine (2018-TH-003). Informed consent was supplied by the participating individual.

### Parasite culture and treatment by MTZ

*T. vaginalis* were cultured in modified hepar-peptone-glucose medium supplemented with 10% heat-inactivated fetal calf serum, 100 U/ml penicillin, and 100 µg/ml streptomycin and maintained at 37 °C. Only the logarithmic-phase parasites were used in this work. Before RNA-seq, the MTZ susceptibility of *T. vaginalis* was determined by microscopy. *T. vaginalis*, approximately 2 × 10^5^/ml in 200 µl, was incubated with different concentrations (from 200 to 0 µg/ml) of MTZ in 96-well plates for 24 h. The drug concentration at which no viable parasites were observed after trypan blue staining was defined as the MLC. The MTZ IC_50_ of *T. vaginalis* was determined after treatment for 3 h in vitro. The drug concentration at which approximately half of the parasites compared to the control (MTZ = 0 µg/ml) was chosen for electron microscopic observation and RNA-seq.

### Preparation of scanning electron microscopy and transmission electron microscopy

Approximately 5 × 10^7^*T. vaginalis* trophozoites at the logarithmic phase were collected and fixed in 2.5% glutaraldehyde. The parasites were postfixed for 2 h at room temperature in 1% OsO4. Then, they were dehydrated in graded ethanol and isoamyl acetate and dried with a critical point dryer. The specimens were attached to metallic stubs using carbon stickers and sputter-coated with gold for 30 s. Images were collected with a scanning electron microscope (Regulus 8100, HITACHI).

For transmission electron microscopy, *T. vaginalis* fixed in 2.5% glutaraldehyde were first preembedded in agarose and then postfixed in 1% OsO4 for 2 h at room temperature. After being dehydrated in graded ethanol and acetone, they were embedded in EMBed 812. The resin blocks were cut to 60–80 nm, and the tissues were fished onto 150-mesh cuprum grids with formvar film. Then, the sections were stained with 2% uranium acetate and 2.6% lead citrate. Images were finally collected with a transmission electron microscope (HT7700, HITACHI).

### RNA extraction and cDNA library preparation for illumina sequencing

The parasite materials were maintained with TRIzol at -80 °C until RNA extraction. Sequencing was performed by the Frasergen Corporation (Wuhan, China). After RNA contamination and degradation were monitored on 1.5% agarose gels, RNA purity and concentration were measured with a NanoPhotometer® spectrophotometer (IMPLEN, CA, USA) and Qubit® RNA Assay Kit in Qubit® 3.0 Fluorometer (Life Technologies, CA, USA), respectively. Then, RNA integrity was assessed by using the RNA Nano 6000 Assay Kit of the Agilent Bioanalyzer 2100 system (Agilent Technologies, CA, USA).

Qualified RNA was submitted to generate sequencing libraries using the NEBNext® UltraTM RNA Library Prep Kit for Illumina® (NEB, USA) following the manufacturer’s instructions and index codes. Briefly, mRNA was first gathered from the total RNA with poly-T oligo-attached magnetic beads. Then, mRNA was fragmented with divalent cations under elevated temperature in NEB Next First Strand Synthesis Reaction Buffer (5×). By using random hexamer primers and M-MuLV Reverse Transcriptase, first-strand cDNA was synthesized, which was followed by the synthesis of second-strand cDNA using DNA Polymerase I and RNase H. After double-stranded cDNA was purified by AMPure XP beads, USER enzyme was used to degrade the second strand cDNA containing U. The remaining purified double-stranded cDNA was processed with an end-repair reaction, the addition of a single ‘A’ base and adapter ligation. To select and purify cDNA fragments with the right length, PCR and AMPure XP beads (Beckman Coulter, Beverly, USA) were applied to obtain the final cDNA library. The qualified library assessed by the Agilent Bioanalyzer 2100 System was finally sequenced using the Illumina HiSeq2000.

### RNA-seq data analysis

For quality control of the sequencing data, the software FraserQC (v1.2), an in-house software developed by Frasergen Co. Ltd. (Wuhan, China), was used. Clean data (clean reads) were selected from the raw data after a series of routine processes. For read alignment, the *T. vaginalis* genome obtained by *de novo* sequencing on the PacBio Sequel platform (Pacific Biosciences of California, Menlo Park, CA, USA) conducted by our laboratory was chosen as the reference genome by using Tophat2 (v2.1.1) [43] and Bowtie2 (v2.2.2) [44] with default parameters. The expression levels of genes were quantified using the software package RSEM (RNASeq by Expectation Maximization v1.3.0) [45]. After FPKM transformation [46], screening of DEGs with the EdgeR (v3.6.8) package [47] was performed based on the following criteria: fold change (FC = condition 2/condition 1 for a gene) ≥ 2 and FDR < 0.05. The upregulated DEGs met the requirements of FDR < 0.05 and Log_2_ FC > 1, and the downregulated DEGs met the criteria of FDR < 0.05 and Log_2_ FC< -1. To visualize the distribution of FC and FDR values of all genes between the two groups, MA plot and volcano plot were drawn. Then, the numbers of upregulated and downregulated DEGs were sent for statistical analysis and heatmap drawing of the known gene expression pattern clusters.

### GO and KEGG enrichment analysis

After screening out target DEGs, Gene Ontology (GO) enrichment analysis was accomplished using GOseq (v1.22) based on Wallenius noncentral hypergeometric distribution, which contains three ontologies: molecular function, biological process, and cellular component. Kyoto Encyclopedia of Genes and Genomes (KEGG) pathway analysis of DEGs was performed in KOBAS (v3.0) [48–50]. For both GO enrichment and KEGG pathway analysis, FDR ≤ 0.05 was considered significantly enriched.

### Quantification and region-based annotation of SNPs and indels

The Picard tools version 1.96 were used to sort the bam files obtained by comparing them with the reference genomes. After duplication removal, SNPs and indels were called using SAMtools version 0.1.19 and Genome Analysis Toolkit (GATK) version 3.7. Finally, the obtained SNPs and indels with high quality were sent for region-based annotation using ANNOVAR version 2.1.

### Quantitative real-time PCR (RT‒qPCR)

The parasite materials and RNA extraction were the same as those used for RNA-Seq. Reverse transcription and qPCR were conducted using the PrimeScript™ RT reagent Kit with gDNA Eraser (Perfect Real Time) and TB Green® Premix Ex Taq™ II (Tli RNaseH Plus) kit according to the manufacturer’s protocols (Takara, Dalian, China). Briefly, after the genomic DNA removal reaction, total RNA was reverse transcribed to cDNA, which was used as the template for the following qPCRs. The reaction systems and conditions of RT‒qPCR were all in accordance with the manufacturer’s suggestion on a CFX96 Real-Time PCR Detection System (Bio-Rad, USA). Melting curve analysis was performed to validate specific amplification. The primers used for RT‒qPCR were designed with primer-BLAST. The actin gene was used as the reference gene for normalization, and the relative expression was calculated with the 2^−∆∆Ct^ method. Three technical replicates and two negative controls without template were set up for each biological replicate.

### Statistical analysis

The plots and statistical analysis of the MLC and IC_50_ of MTZ against *T. vaginalis*, the statistics for distribution of SNP and indel, the relative quantification by RT-qPCR and the correlation between RNA-Seq with RT-qPCR were all performed using GraphPad Prism 8.0.2. The determinations were expressed as the means ± standard deviations (SDs). Correlation analysis was performed with linear regression. SNP and indels with significant differences between groups were analyzed by T-test (F test, P>0.05), or Welch’s t test (F test, P < 0.05) in GraphPad Prism 8.0.2.

## Electronic supplementary material

Below is the link to the electronic supplementary material.

Supplementary Material 1

Supplementary Material 2

Supplementary Material 3

Supplementary Material 4

Supplementary Material 5

Supplementary Material 6

Supplementary Material 7

Supplementary Material 8

Supplementary Material 9

Supplementary Material 10

## Data Availability

The clean data sets for sequencing presented in this study were uploaded into the Sequence Read Archive (SRA) in online repositories. The BioProject accession code PRJNA849385 can be found in NCBI through the website https://www.ncbi.nlm.nih.gov/sra/PRJNA849385.

## Abbreviations

MTZ: Metronidazole
DEGs: Differentially expressed gene
WHO: World Health Organization
PFOR: Pyruvate:ferredoxin oxidoreductase
Fd: Ferredoxin
SNP: Single nucleotide polymorphism
Indels: Insertions-deletions
MLC: Minimum lethal concentration
FPKM: Fragments per kilobase per million bases
FC: Fold change
FDR: False discovery rate
GO: Gene ontology
KEGG: Kyoto Encyclopedia of Genes and Genomes
SRA: Sequence read archive
Fld: Flavodoxin
NADPH: Nicotinamide adenine dinucleotide phosphate
TrxR: Thioredoxin reductase
NTR: Nitroreductase
Trx: Thioredoxin
TrxP: Thioredoxin peroxidase
