# Supplementary material for: Transcriptional profile of Trichomonas vaginalis in response to metronidazole
Source: BMC Genomics. 2023 Jun 12;24:318. doi: 10.1186/s12864-023-09339-9 (PMC10262402; doi:10.1186/s12864-023-09339-9)
Supplement: Supplementary file 1 — Supplementary Material 1 [file 12864_2023_9339_MOESM1_ESM.docx]

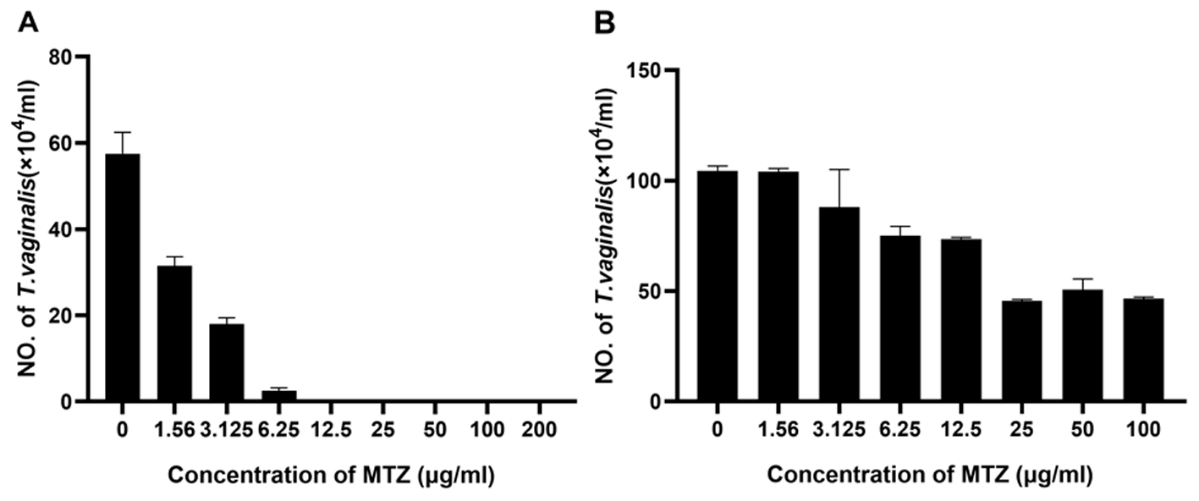


**Supplementary Figure S1**. Validation of MTZ susceptibility and IC_50_ of TV-THS1. **(A)** The MTZ susceptibility of *T. vaginalis* was determined by microscopy after treatment for 24 h *in vitro*. The drug concentration at which no viable parasites were observed after trypan blue staining was defined as the minimum lethal concentration. **(B)** The MTZ IC_50_ of *T. vaginalis* was determined after treatment for 3 hrs *in vitro*. The drug concentration that was approximately half of the parasites compared to the control (MTZ=0 μg/ml) was chosen for [RNA-seq](javascript:;).
