# Supplementary material for: Transcriptional profile of Trichomonas vaginalis in response to metronidazole
Source: BMC Genomics. 2023 Jun 12;24:318. doi: 10.1186/s12864-023-09339-9 (PMC10262402; doi:10.1186/s12864-023-09339-9)
Supplement: Supplementary file 2 — Supplementary Material 2 [file 12864_2023_9339_MOESM2_ESM.docx]

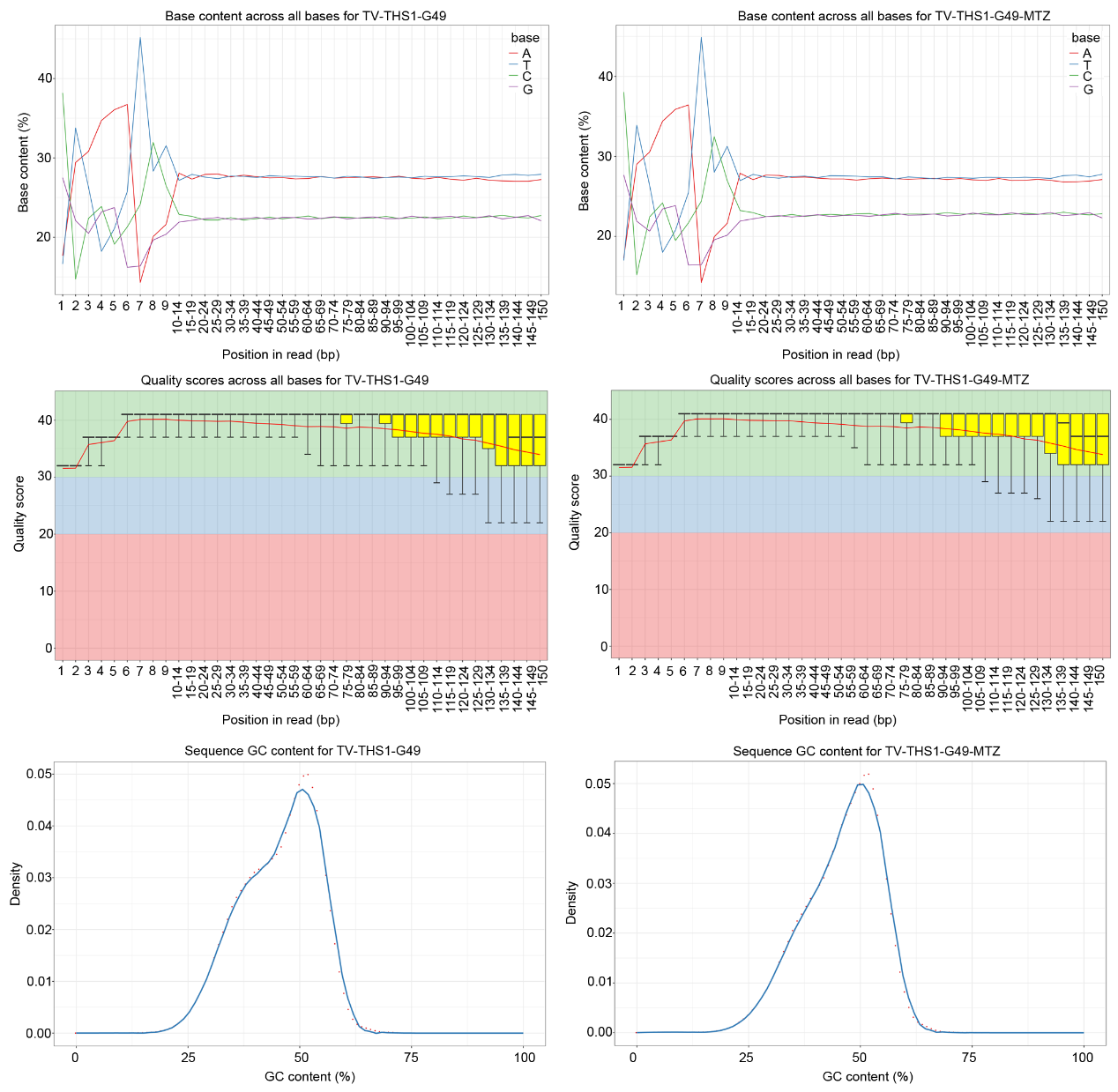


**Supplementary Figure S2.** Quality assessment of sequencing data. The quality of sequencing data was assessed by base content across all bases, quality scores across all bases, and GC content.
