# Supplementary material for: Transcriptional profile of Trichomonas vaginalis in response to metronidazole
Source: BMC Genomics. 2023 Jun 12;24:318. doi: 10.1186/s12864-023-09339-9 (PMC10262402; doi:10.1186/s12864-023-09339-9)
Supplement: Supplementary file 3 — Supplementary Material 3 [file 12864_2023_9339_MOESM3_ESM.docx]

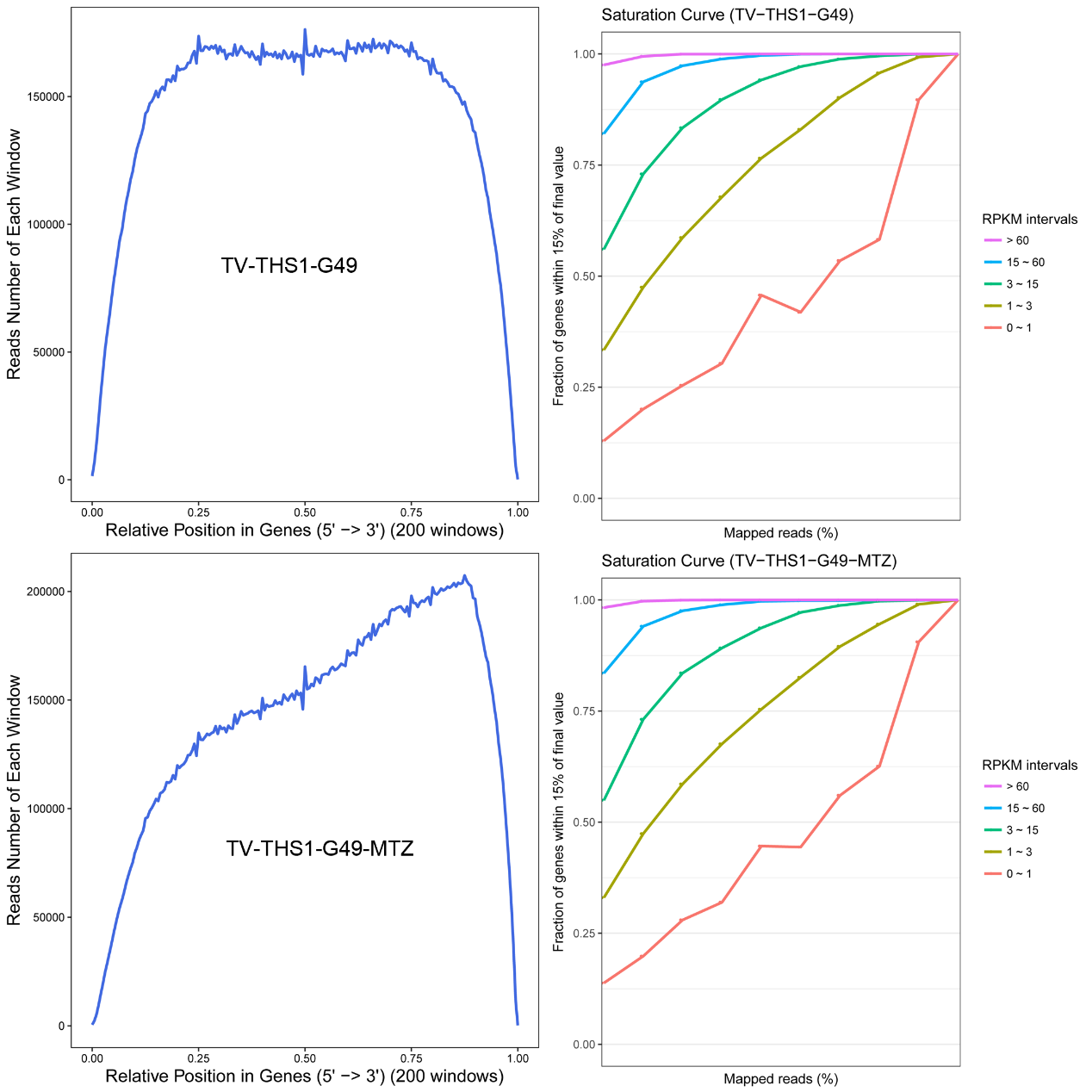


**Supplementary Figure S3.** Assessment of integral quality of RNA-seq. The integral quality of RNA-seq was assessed by random distribution of RNA sequencing and saturability analysis.
