# Supplementary material for: Transcriptional profile of Trichomonas vaginalis in response to metronidazole
Source: BMC Genomics. 2023 Jun 12;24:318. doi: 10.1186/s12864-023-09339-9 (PMC10262402; doi:10.1186/s12864-023-09339-9)
Supplement: Supplementary file 4 — Supplementary Material 4 [file 12864_2023_9339_MOESM4_ESM.docx]

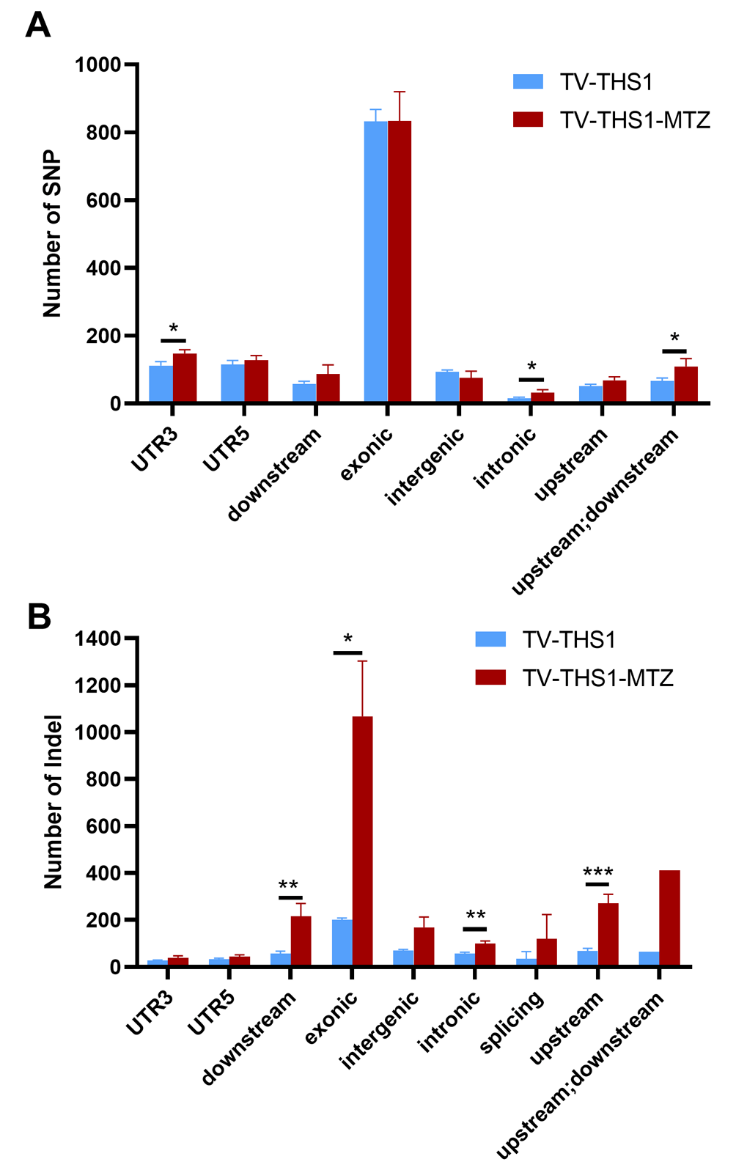


**Supplementary Figure S4.** The genetic distribution and quantity of SNPs and indels. The number of SNPs (A) and indels (B) in different gene regions of *T. vaginalis* untreated or treated with MTZ. UTR3: the number of SNPs or indels in the 3’UTR; UTR5: the number of SNPs or indels in the 5’UTR; downstream: the number of SNPs or indels in the 3 kb region of downstream from the transcription termination site; exonic: the number of SNPs or indels in the exon region; intergenic: the number of SNPs or indels in the intergene region; intronic: the number of SNPs or indels in the intron region; upstream: the number of SNPs or indels in 3 kb region of upstream from transcription start site; [upstream; downstream]: the number of SNPs or indels in both upstream region and downstream region; splicing: the number of indels in 10 bp region of splicing junction. Statistically significant differences between the untreated and MTZ-treated groups are marked as *, *P<* 0.05; **, *P <* 0.01; ***, *P* < 0.001.
