# Supplementary material for: Transcriptional profile of Trichomonas vaginalis in response to metronidazole
Source: BMC Genomics. 2023 Jun 12;24:318. doi: 10.1186/s12864-023-09339-9 (PMC10262402; doi:10.1186/s12864-023-09339-9)
Supplement: Supplementary file 5 — Supplementary Material 5 [file 12864_2023_9339_MOESM5_ESM.docx]

**Supplementary Table S1.** Mapping of reads to the reference genome and reference gene

| **Mapping of reads to**  **the reference genome** | **Total**  **Reads** | **Total mapping**  **rate** | **Left reads**  **mapped** | **Left reads**  **multiple mapped** | **Right reads**  **mapped** | **Right reads**  **multiple mapped** |
| --- | --- | --- | --- | --- | --- | --- |
| **TV-THS1-G49** | 36,612,352 | 32,858,430(89.7%) | 16,520,427(90.2%) | 1,556,665(9.4%) | 16,338,003(89.2%) | 1,510,008(9.2%) |
| **TV-THS1-G50** | 43,767,744 | 39,254,290(89.7%) | 19,701,214(90.0%) | 1,836,968(9.3%) | 19,553,076(89.3%) | 1,793,512(9.2%) |
| **TV-THS1-G51** | 37,583,390 | 33,742,247(89.8%) | 16,954,209(90.2%) | 1,595,205(9.4%) | 16,788,038(89.3%) | 1,546,662(9.2%) |
| **TV-THS1-G49-MTZ** | 41,163,792 | 35,913,253(87.2%) | 18,073,607(87.8%) | 2,254,043(12.5%) | 17,839,646(86.7%) | 2,198,529(12.3%) |
| **TV-THS1-G50-MTZ** | 42,639,490 | 37,376,961(87.7%) | 18,757,469(88.0%) | 2,331,039(12.4%) | 18,619,492(87.3%) | 2,291,725(12.3%) |
| **TV-THS1-G51-MTZ** | 41,870,320 | 36,117,574(86.3%) | 18,192,301(86.9%) | 2,360,373(13.0%) | 17,925,273(85.6%) | 2,305,603(12.9%) |
