# Supplementary material for: Transcriptional profile of Trichomonas vaginalis in response to metronidazole
Source: BMC Genomics. 2023 Jun 12;24:318. doi: 10.1186/s12864-023-09339-9 (PMC10262402; doi:10.1186/s12864-023-09339-9)
Supplement: Supplementary file 6 — Supplementary Material 6 [file 12864_2023_9339_MOESM6_ESM.docx]

**Supplementary Table S2.** Statistics of the number of expressed genes at different expression levels

| **Sample** | **Expressed_Gene** | **Total_Gene** | **0** | **0~1** | **1~3** | **3~15** | **15~60** | **>60** |
| --- | --- | --- | --- | --- | --- | --- | --- | --- |
| **TV-THS1-G49** | 22,969 | 33,867 | 10,898(32.18%) | 3,807(11.24%) | 4,050(11.96%) | 7,910(23.36%) | 4,210(12.43%) | 2,992(8.83%) |
| **TV-THS1-G50** | 23,160 | 33,867 | 10,707(31.61%) | 4,055(11.97%) | 4,427(13.07%) | 7,990(23.59%) | 3,873(11.44%) | 2,815(8.31%) |
| **TV-THS1-G51** | 23,496 | 33,867 | 10,371(30.62%) | 3,373(9.96%) | 4,514(13.33%) | 8,296(24.50%) | 4,258(12.57%) | 3,055(9.02%) |
| **TV-THS1-G49-MTZ** | 22,194 | 33,867 | 11,673(34.47%) | 7,456(22.02%) | 4,637(13.69%) | 5,195(15.34%) | 2,387(7.05%) | 2,519(7.44%) |
| **TV-THS1-G50-MTZ** | 22,867 | 33,867 | 11,000(32.48%) | 7,378(21.79%) | 4,805(14.19%) | 5,476(16.17%) | 2,629(7.76%) | 2,579(7.62%) |
| **TV-THS1-G51-MTZ** | 23,370 | 33,867 | 10,497(30.99%) | 7,151(21.11%) | 5,025(14.84%) | 5,784(17.08%) | 2,782(8.21%) | 2,628(7.76%) |
