# Supplementary material for: Transcriptional profile of Trichomonas vaginalis in response to metronidazole
Source: BMC Genomics. 2023 Jun 12;24:318. doi: 10.1186/s12864-023-09339-9 (PMC10262402; doi:10.1186/s12864-023-09339-9)
Supplement: Supplementary file 9 — Supplementary Material 9 [file 12864_2023_9339_MOESM9_ESM.docx]

**Supplementary Table S5.** The significantly enriched pathways except for top 20 of downregulated DEGs by KEGG

| **Pathway Hierarchy1** | **Pathway Hierarchy2** | **KEGG Pathway** | **Gene**  **Number** | **Background**  **number** | **Rich**  **factor** | **Q-Value** |
| --- | --- | --- | --- | --- | --- | --- |
| **Organismal Systems** | Sensory system | Inflammatory mediator regulation of TRP channels | 56 | 162 | 0.346 | 1.68E-02 |
|  | Nervous system | Long-term potentiation | 64 | 188 | 0.340 | 1.40E-02 |
|  |  | Long-term depression | 33 | 86 | 0.384 | 1.81E-02 |
|  | Immune system | T cell receptor signaling pathway | 38 | 94 | 0.404 | 3.82E-03 |
|  |  | Chemokine signaling pathway | 37 | 93 | 0.398 | 6.04E-03 |
|  |  | Toll-like receptor signaling pathway | 22 | 54 | 0.407 | 3.35E-02 |
|  |  | Fc epsilon RI signaling pathway | 22 | 55 | 0.400 | 4.03E-02 |
|  |  | Th17 cell differentiation | 21 | 53 | 0.396 | 4.89E-02 |
|  | Excretory system | Vasopressin-regulated water reabsorption | 30 | 80 | 0.375 | 3.60E-02 |
|  | Environmental adaptation | Circadian rhythm - plant | 23 | 47 | 0.489 | 2.00E-03 |
|  |  | Circadian rhythm | 22 | 43 | 0.512 | 1.27E-03 |
|  | Endocrine system | Thyroid hormone signaling pathway | 64 | 151 | 0.424 | 5.45E-05 |
|  |  | Progesterone-mediated oocyte maturation | 53 | 137 | 0.387 | 1.49E-03 |
|  |  | Adipocytokine signaling pathway | 51 | 140 | 0.364 | 8.03E-03 |
|  |  | Prolactin signaling pathway | 24 | 55 | 0.436 | 9.68E-03 |
|  | Development | Dorso-ventral axis formation | 11 | 21 | 0.524 | 2.87E-02 |
|  | Aging | Longevity regulating pathway - mammal | 68 | 189 | 0.360 | 2.45E-03 |
|  |  | Longevity regulating pathway - multiple species | 58 | 176 | 0.330 | 4.00E-02 |
| **Genetic Information Processing** | Translation | Ribosome biogenesis in eukaryotes | 65 | 158 | 0.411 | 1.01E-04 |
|  | Transcription | Basal transcription factors | 28 | 49 | 0.571 | 5.45E-05 |
| **Environmental Information Processing** | Signal transduction | FoxO signaling pathway | 63 | 180 | 0.350 | 7.95E-03 |
|  |  | Ras signaling pathway | 61 | 189 | 0.323 | 4.89E-02 |
|  |  | Wnt signaling pathway | 57 | 143 | 0.399 | 4.07E-04 |
|  |  | ErbB signaling pathway | 50 | 113 | 0.442 | 1.01E-04 |
|  |  | Jak-STAT signaling pathway | 43 | 97 | 0.443 | 2.83E-04 |
|  |  | HIF-1 signaling pathway | 42 | 123 | 0.341 | 4.94E-02 |
|  |  | Sphingolipid signaling pathway | 41 | 118 | 0.347 | 4.17E-02 |
|  |  | MAPK signaling pathway - fly | 39 | 104 | 0.375 | 1.40E-02 |
|  |  | NF-kappa B signaling pathway | 33 | 69 | 0.478 | 2.89E-04 |
|  |  | Hedgehog signaling pathway | 27 | 53 | 0.509 | 3.41E-04 |
| **Cellular Processes** | Transport and catabolism | Autophagy - yeast | 65 | 201 | 0.323 | 4.03E-02 |
|  |  | Mitophagy - yeast | 48 | 108 | 0.444 | 1.17E-04 |
|  |  | Mitophagy - animal | 38 | 99 | 0.384 | 1.02E-02 |
|  |  | Autophagy - other eukaryotes | 38 | 103 | 0.369 | 2.04E-02 |
|  | Cellular community eukaryotes | Gap junction | 55 | 141 | 0.390 | 9.78E-04 |
|  |  | Adherens junction | 47 | 113 | 0.416 | 5.17E-04 |
|  | Cell growth and death | Cell cycle - yeast | 42 | 122 | 0.344 | 4.49E-02 |
