# Supplementary material for: Transcriptional profile of Trichomonas vaginalis in response to metronidazole
Source: BMC Genomics. 2023 Jun 12;24:318. doi: 10.1186/s12864-023-09339-9 (PMC10262402; doi:10.1186/s12864-023-09339-9)
Supplement: Supplementary file 10 — Supplementary Material 10 [file 12864_2023_9339_MOESM10_ESM.docx]

**Supplementary Table S6.** Primer information of the selected genes for qPCR

| **GenBank Accession Numbers** | **Gene Annotation** | **Primer sequence (5’ to 3’)** | **Amplicon length (bp)** |
| --- | --- | --- | --- |
| EAY02588.1 | Pyruvate:ferredoxin oxidoreductase E | F: GAGGCCATCTGCAAGAACCT  R: ATGGCTGTCTCTGGGTTGTG | 80 |
| EAY19262.1 | 4Fe-4S binding domain containing protein | F: GCTCCATCTACAGGCAAGCA  R: GTAACTTTGTGGCCCCGAGA | 95 |
| EAY22303.1 | 4Fe-4S binding domain containing protein | F: AATTGAACCACGCTGCTTGC  R: CCGAAGTGGCCGAATGTTTG | 103 |
| EAY04700.1 | Thioredoxin reductase | F: ACAAGACAGGCGAGACACAG  R: CCTGGGCATCTGTCTCAAGG | 108 |
| EAY20871.1 | Flavodoxin-like fold family protein | F: AGAACGCCAAGGTCCTCATC  R: CTGCCTCCAAAGCAAGGGTT | 90 |
| EAX97338.1 | Flavodoxin-like fold family protein | F: GGTTCTTAGGTCTCCCAGCAG  R: TGCCTCCAAGTGTGTAACCC | 81 |
| EAY00354.1 | Flavodoxin-like fold family protein | F: CAGGTGTTGCCCCAATGTTC  R: TTGCGCCGTAAACGTCAAGA | 129 |
| EAY15747.1 | Flavodoxin-like fold family protein | F: GTATGGCACAGGTGGATGCT  R: GTCTGCGTAGAACTGGCCTT | 103 |
| XP_001307810.1 | Flavodoxin-like fold | F: GCGGTGTTGAATCTGTTGGC  R: TTACAAGCGAGGCACGACTG | 88 |
| XP_001322702.1 | Flavodoxin-like fold | F: TCCACCAATGTCACAAGGCA  R: GACTTGATGGCCTTTGTGCTC | 82 |
| XP_001317528.1 | Flavodoxin-like fold | F: ACACGAACCAGGCCAATTCT  R: CCAACAAATGTCATGCAGGCT | 81 |
| XP_001317526.1 | Flavodoxin-like fold | F: CGGAAGTGGAGGACTTTGTCA  R: AGAATTGGCCTGGTTCGTGT | 92 |
| XP_001306204.1 | Flavodoxin-like fold | F: CCATGGGGTGCAAAGAAGTA  R: CTGGCAGAGACCACCACTG | 117 |
| XP_001319426.1 | Flavodoxin-like fold | F: TCCCAATCTGGTGGATGGGT  R: TGCCATCACCGTTCCAAAGT | 96 |
| TVAG_534990 | Actin | F：TCACAGCTCTTGCTCCACCA  R：AAGCACTTGCGGTGAACGAT | 175 |
